# 564. SARS-CoV-2 Ferritin Nanoparticle Vaccines Elicit Broad SARS Coronavirus Immunogenicity

**DOI:** 10.1093/ofid/ofab466.762

**Published:** 2021-12-04

**Authors:** M G Joyce, Wei-Hung Chen, Rajeshwer Sankhala, Agnes Hajduczki, Paul Thomas, Elizabeth Martinez, Caroline Peterson, Mangala Rao, Kayvon Modjarrad

**Affiliations:** 1 Henry M. Jackson Foundation/Walter Reed Army Institute of Research, Silver Spring, Maryland; 2 Military HIV Research Program, Silver Spring, Maryland; 3 Walter Reed Army Institute of Research, Silver Spring, Maryland

## Abstract

**Background:**

The zoonotic emergence of SARS-CoV-2 quickly developed into a global pandemic. Multiple vaccine platforms have been advanced to clinical trials and emergency use authorization. The recent emergence of SARS-CoV-2 virus variants with Spike receptor-binding domain (RBD) and N-terminal domain (NTD) mutations, highlights the need for next-generation vaccines that can elicit immune responses that are resilient against Spike mutations.

**Methods:**

Using a structure-based vaccine design approach, we developed multiple optimized SARS-CoV-2 nanoparticle immunogens that recapitulate the structural and antigenic profile of the SARS-CoV-2 prefusion spike. We assessed these immunogens in murine immunogenicity studies and in a K18-hACE2 transgenic mouse model with a SARS-CoV-2 challenge. Immune sera from vaccinated mice were assessed for SARS-CoV-2 binding, and neutralization against SARS-CoV-2, variants of concern, and the heterologous SARS-CoV-1 virus.

**Results:**

In combination with a liposomal-saponin based adjuvant (ALFQ), these immunogens induced robust binding, ACE2-inhibition, and authentic virus and pseudovirus neutralization. A Spike-Ferritin nanoparticle (SpFN) vaccine elicited neutralizing ID50 titers >10,000 after a single immunization, while RBD-Ferritin (RFN) nanoparticle immunogens elicited ID50 titer values >10,000 values after two immunizations. Purified antibody from SpFN- or RFN-immunized mice was transfused into K18-ACE2 transgenic mice and challenged with a high-dose SARS-CoV-2 virus stock. In order to understand the breadth of vaccine-elicited antibody responses, we analyzed SpFN- and RBD-FN-immunized animal sera against a set of heterologous SARS-CoV-2 RBD variants and SARS-CoV RBD. High binding titers with ACE2-blocking activity were observed against SARS-CoV-2 variants and the heterologous SARS-CoV-1 RBD. Furthermore, both SpFN- and RFN-immunized animal sera showed SARS-CoV-1 neutralizing ID50 titers of >2000.

**Conclusion:**

These observations highlight the importance of SARS-CoV-2 neutralizing antibody levels in providing protection against emerging SARS-like coronaviruses and provide a robust platform for pandemic preparedness.

Structure-based design enables development of a SARS-CoV-2 nanoparticle immunogen.

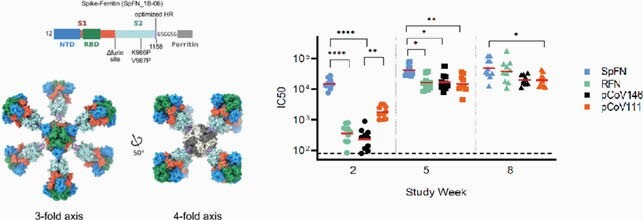

**Disclosures:**

**All Authors**: No reported disclosures

